# Factors Influencing Tissue Cyst Yield in a Murine Model of Chronic Toxoplasmosis

**DOI:** 10.1128/iai.00566-22

**Published:** 2023-06-26

**Authors:** Cortni A. Troublefield, Joy S. Miracle, Robert D. Murphy, Ryan W. Donkin, Anthony P. Sinai

**Affiliations:** a Department of Microbiology, Immunology, and Molecular Genetics, University of Kentucky College of Medicine, Lexington, Kentucky, USA; b Department of Molecular and Cellular Biochemistry, University of Kentucky College of Medicine, Lexington, Kentucky, USA; University of California Davis

**Keywords:** *Toxoplasma gondii*, bradyzoite, tissue cyst

## Abstract

Recent advances into the unique biology of *Toxoplasma* tissue cysts and the bradyzoites they house necessitate optimization of tissue cyst recovery from infected mouse brains. Here, we present data from 83 tissue cyst purifications of Type II ME49 tissue cysts in CBA/J mice performed over a period of 3 years. The effects of infection with both tissue culture tachyzoites as well as *ex vivo* tissue cysts were assessed. Significant mortality was restricted to tachyzoite infections with female mice being more susceptible. Infection with tissue cysts was associated with both lower overall symptomology and mortality, exhibiting no sex bias. Cumulatively, host sex did not impact overall tissue cyst yields, although tachyzoite-initiated infections generated significantly higher yields compared to tissue cyst-initiated infections. Notably, serial passage of tissue cysts was accompanied with a decreasing trend for subsequent cyst recovery. The time of tissue cyst harvest, a potential reflection of bradyzoite physiological state, had no significant impact on subsequent cyst yield at the selected time points. In aggregate, these data reveal the considerable heterogeneity associated with tissue cyst yield, making the design of adequately powered experiments critical. This is particularly the case for drug studies where overall tissue cyst burden is currently the primary and often sole metric of efficacy, as the data presented here demonstrate that cyst recovery between preparations of untreated animals can mirror and even exceed the reported effects of drug treatment.

## INTRODUCTION

Toxoplasma gondii is an important opportunistic infection in the context of HIV-AIDS and other immunosuppressive conditions (1, 2). Transmission of this parasite is mediated by two distinct encysted forms: the oocysts shed at the end of the sexual cycle in the feces of the definitive feline host (3, 4), and the tissue cysts formed with the establishment of chronic infection in all vertebrate hosts (5). The tropism of tissue cysts to the central nervous system and muscle tissues provides a mechanism of transmission in the act of carnivory (6, 7). Indeed, consumption of raw or undercooked meat contaminated with tissue cysts is the primary mechanism of transmission to humans, contributing to T. gondii being a significant agent of foodborne infection (6–8).

Despite their central role in transmission, little is known about the basic biology of tissue cysts (5). What is known is that the tissue cyst represents a genetically clonal, though physiologically heterogenous, community of slow-growing bradyzoite forms of the parasite (5). Bradyzoites have long been viewed as dormant forms, a position that was challenged by our work showing that tissue cysts derived from infected mouse brains contain bradyzoites exhibiting metabolic activity, including the capacity to replicate by endodyogeny (9). This critical finding has triggered a renewed interest in bradyzoite biology because it provides a window into potential drug treatment for a form that remains refractory to currently approved drugs (10).

The development of new treatments targeting encysted bradyzoites will fundamentally depend on efficient means of generating tissue cysts *in vivo* for further functional analyses (10–13). Here, we build on our earlier work (9, 14) to identify factors influencing tissue cyst yields from infected brains in a murine model of experimental toxoplasmosis. Seeking to optimize the parameters contributing to tissue cyst recovery from the infected brain, we assessed the effects of sex, infection source, serial passage of tissue cysts between mice, and the relative time of harvest on subsequent cyst yield. Additionally, we assessed tissue cyst yields based on mean recovery per infected animal while also considering per capita yields accounting for animals that succumbed to infection prior to harvesting. The latter category proved to represent a significant proportion of animals infected with tissue culture-derived tachyzoites.

The data presented here indicate that tissue cyst yields are impacted by multiple factors, necessitating consideration particularly in the interpretation of drug study outcomes. Currently, drug efficacy is exclusively evaluated based on tissue cyst recovery, the sensitivity of which is limited to under 2 orders of magnitude. Thus, even the most effective experimental drugs which reduce recovery by 80% to 90% (10–13, 15, 16) fall within the range of cyst recovery in the absence of any drug treatment (5, 9) (data presented in this study). We therefore must reassess overall approaches to understanding chronic infection and focus on the level of encysted bradyzoites, appreciating the heterogeneity of activity within the tissue cyst. The development of these methods, several of which are under way in our laboratory, will greatly benefit from the optimization of tissue cyst yield, considering the factors investigated here and other factors that remain to be identified.

## RESULTS

### Generation of a Type II ME49ΔHXGPRT line.

In this study, we generated a Type II ME49ΔHXGPRT line to establish the characteristics that constitute a parental (WT, wild-type) line for subsequent studies targeting specific genes, which are ongoing and beyond the scope of the current study. Targeted disruption of the hypoxanthine-xanthine guanine phosphoribosyl transferase gene (TgME49_200320) has been shown to have no impact on growth both in culture and *in vivo* (acute infection) (17–19). Loss of TgHXGPRT can be positively selected for using the subversive substrate 6-thioxanthine (6-TX) (17, 19). Importantly, restoration of activity in ΔTgHXGPRT lines allows for positive selection using a combination of mycophenolic acid and xanthine (MPA-X), providing a marker for positive selection in subsequent gene targeting/complementation studies (Fig. S1A in the supplemental material) (17, 19).

To disrupt the HXGPRT locus in the prototypical cyst forming Type II ME49 parasites, we employed a shotgun CRISPR-Cas9 strategy (20, 21), described in Materials and Methods. Drug-selected parasites were cloned by limiting dilution and the disruption of the gene was confirmed by PCR amplification of the locus and sequencing across the mutation lesion (Fig. S1B and C). Importantly, infectivity and growth characteristics assessed by both replication assays at 24 h postinfection and plaque assays were identical to those of the parental WT ME49 line (Fig. S1D). In addition, the selected clonal TgME49ΔHXGPRT line exhibited identical rates of stage conversion relative to wild-type ME49 (Fig. S1E). Finally, this line exhibited no significant difference in the capacity to establish both acute and chronic infection in mice, as detailed extensively below. For these reasons, we designate the TgME49ΔHXGPRT parasite used exclusively in this study as the wild type (WT).

### Differential effects of tachyzoite versus tissue cyst infections on mortality.

Literature reports and our prior experience reveal considerable heterogeneity in the mortality caused by ME49 tachyzoites during the course of acute infection (9, 22). These can range from median lethal doses (LD_50_) of 100 to 10^4^ parasites (administered intraperitoneally [i.p.]), with enhanced virulence selected for by passage in culture. Acute virulence is additionally impacted by mouse genetics which directly or indirectly affect the overall cyst burden (23). In this regard, the CBA/J mouse presents an optimal balance of survival during acute infection and tissue cyst yields in the brain (9, 23). However, the recovery of tissue cysts is inherently highly variable (9, 22) and is influenced by a number of factors, as discussed below.

In our earlier studies, we exclusively used female mice and did not differentiate between tachyzoite- or tissue cyst-initiated infections (9). Here, we examined the effect of sex as a variable as well as the effect of the inoculum on overall survival during the progression of the acute phase and entry into the early chronic phase (days 0 to 28). Animals were monitored daily and at least twice daily once they became symptomatic.

**FIG 1:**
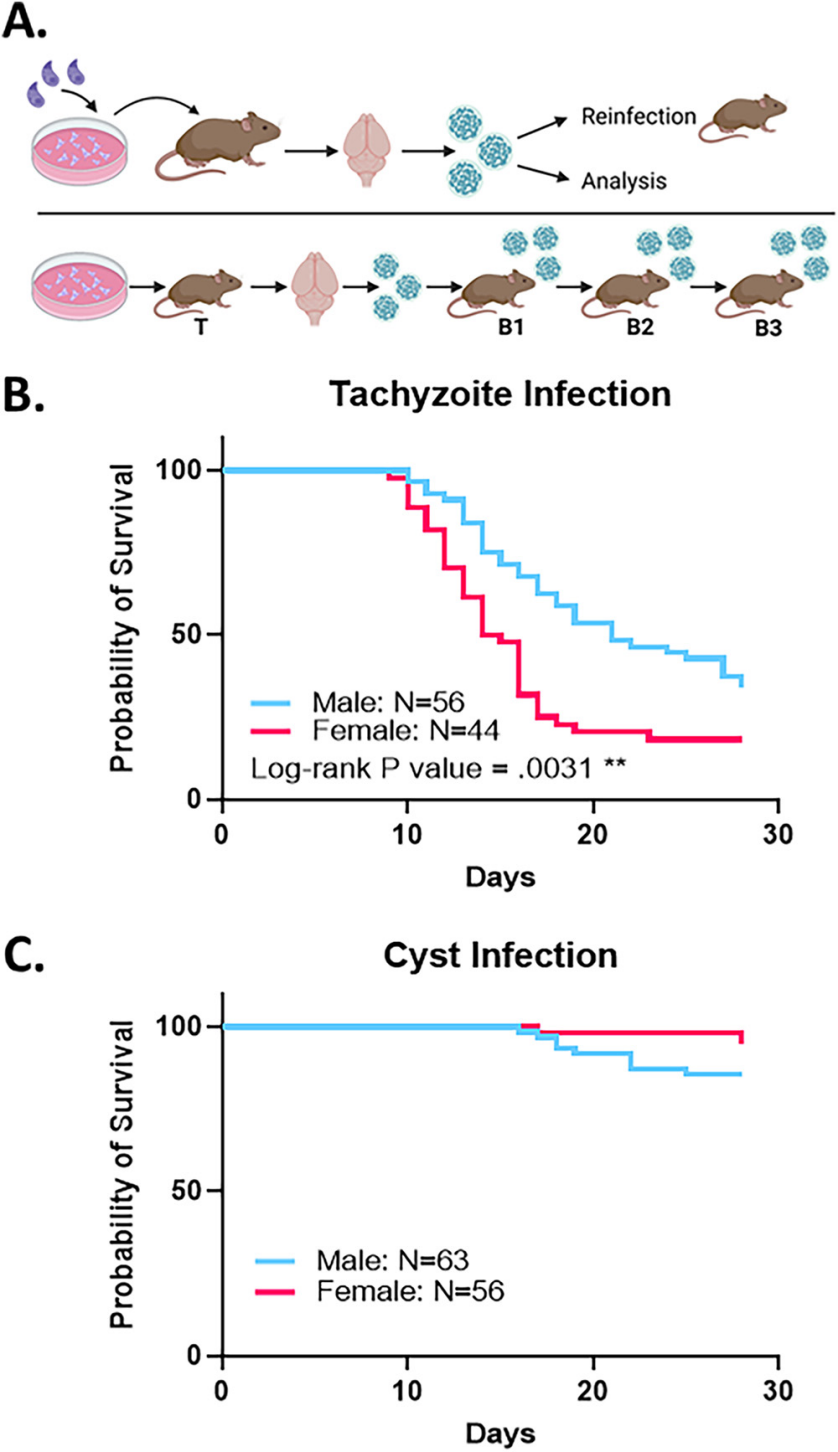
(A) Schematic of the infection model initiated with tissue culture-derived tachyzoites or tissue cysts harvested from infected mouse brains. Purified tissue cysts were used for either serial passage in CBA/J mice or downstream analysis. Row 2: sequential passage of tissue cysts derived from an initial tachyzoite (T) infection to establish the 1st (B1), 2nd (B2), and 3rd (B3) passages. Passages were performed using cysts harvested at 3, 4, 5, 6, or 8 weeks postinfection. (B) Infection of male (blue, *n* = 56) and female (pink, *n* = 44) mice with 100 tissue culture-derived tachyzoites injected i.p. reveal that female mice are significantly more susceptible to infection, as noted by earlier and higher mortality, compared to male mice. (**, log-rank *P* = 0.0031). (C) Low overall mortality was observed following infection of both male (blue, *n* = 63) or female (pink, *n* = 56) mice with 20 *ex vivo* tissue cysts in brain homogenate. Differences in mortality between male and female mice were not statistically significant.

Infection with tachyzoites resulted in significant mortality in mice of both sexes, with female mice exhibiting significantly higher mortality compared to males (Fig. 1B). In contrast, relatively low mortality was observed for both sexes with tissue cyst-initiated infections (Fig. 1C). This finding is meaningful in light of the fact that 20 tissue cysts likely possess an excess of 10^5^ parasites (9), a number at least 3 orders of magnitude greater than those which cause significant mortality in tachyzoite infections.

### Symptomology observed in *T. gondii*-infected animals.

**FIG 2:**
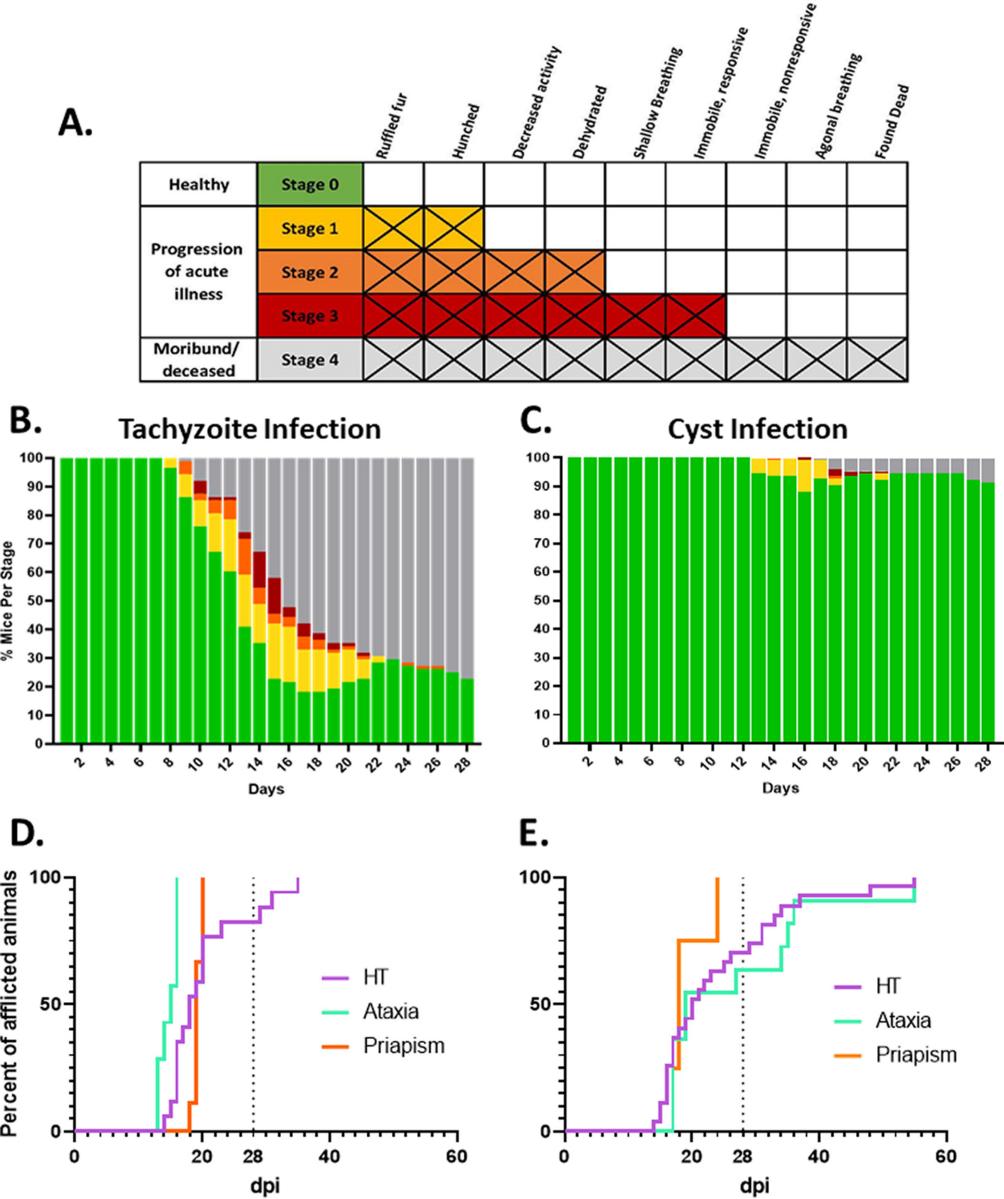
(A) Color-coded body score index rubric based on symptomology associated with the progression of the acute and chronic phases of *Toxoplasma* infection. (B) Progression of symptomology associated with tachyzoite infection through the acute (days 1 to 20) and early chronic phases (days 21 to 28). Symptomology mirrors the mortality profile. Total mice, *n* = 100. (C) Low symptomology and delayed initial onset of symptoms are associated with tissue cyst-initiated infections. Total mice, *n* = 119. (D) Initial appearance of head tilt (purple), ataxia (green), and penile prolapse/priapism (orange) following tachyzoite infection. (E) Initial appearance of head tilt, ataxia, and penile prolapse/priapism in tissue cyst-initiated infections. Dashed line marks day 28 postinfection. Total number of animals displaying head tilt: tachyzoite infection (*n* = 17), cyst infection (*n* = 27). Ataxia: tachyzoite infection (*n* = 7), cyst infection (*n* = 11). Penile prolapse/priapism: tachyzoite infection (*n* = 9), cyst infection (*n* = 4). Images of mice displaying mild, moderate, and severe head tilts are in Fig. S2. Movie S1 shows the diversity of head tilt severity and associated effects on mobility.

The establishment of chronic infection is preceded by the acute phase, within which the extent and timing of symptomology is progressive and variable. As part of our monitoring protocol, we developed a rubric based on a body score index correlated with the severity of symptoms (Fig. 2A). Notably, animals that do not progress past stage 1 and a proportion of animals that enter stage 2 recover and enter chronic infection. In contrast, few animals in stage 3 survive, while none of those in stage 4 survive. Importantly, symptomology is not restricted to the acute phase (days 1 to 20) of infection but can manifest in both the early (up to day 28) and later phases (past day 28) of chronic infection. Symptomology in the chronic infection phase is likely due to spontaneous localized reactivation within the central nervous system.

Consistent with the mortality data, tachyzoite-initiated infections resulted in more rapid onset and severe progression (Fig. 2B) compared to infections initiated with tissue cysts (Fig. 2C). In addition to the classical symptoms and symptom progression/resolution associated with active disease, we noted additional symptoms and conditions that manifested sporadically in a subset of animals. The most frequent of these was the presentation of a “head tilt,” as defined by our veterinary staff. Mice exhibiting a head tilt have their heads cocked to one side and tend to lean in that direction as they move (Fig. S2 and Movie S1). The extent of the head tilt can be mild to severe, with severely afflicted animals moving in circles or on their sides. In these cases, their inability to eat or access water resulted in rapid deterioration in overall condition despite access to wet chow/gel diet on the cage floor. These animals were euthanized based on veterinary assessment. Less severe head tilts often resolved spontaneously. This condition first appears in the course of the acute infection but can also emerge spontaneously in the chronic phase (Fig. 2D and E), and were seen in both tachyzoite (17 cases) and tissue cyst-initiated infections (26 cases), representing 13.1% of all infected animals. There appeared to be no sex-based predisposition for the appearance of a head tilt.

Another symptom observed in a subset of animals was the development of ataxia, noted by clear deficits in the functioning of 1 or more limbs impacting the ability to move and feed. The onset of ataxia, when evident, was more rapid in tachyzoite-initiated infections (Fig. 2D) than in tissue cyst-initiated infections. Interestingly, all cases of ataxia in tachyzoite-initiated infections coincided with the peak of the acute infection, while roughly half of ataxic animals in tissue cyst-initiated infections presented symptoms well into the chronic phase (day >28). Overall, 5.5% of animals exhibited ataxia.

The final atypical symptom appearing with some frequency was the development of what the veterinary staff identified as a penile prolapse and/or priapism. This condition, restricted to male mice, results in sustained penile tumescence in the absence of any sexual activity/arousal. In this state, the animals experienced difficulty with urination, penile irritation, and infection. In the majority of priapism cases, the condition of afflicted animals deteriorated rapidly, necessitating euthanasia upon veterinary advice. A total of 13 out of 168 (7.7%) male mice experienced penile prolapses and all cases were associated with onset late in the acute phase for both tachyzoite- (9/68, 13.2%) and cyst-initiated (4/100, 4%) infections.

Consistent with emerging reports (24), infected mice presented with observed seizures during both the acute and chronic phases of infection. In several instances, the observed seizures were severe enough to warrant euthanasia. It is possible that the otherwise healthy-looking infected animals that were found dead during routine daily inspections had succumbed to a seizure.

### Effect of mouse sex and infection type on tissue cyst recovery.

**FIG 3:**
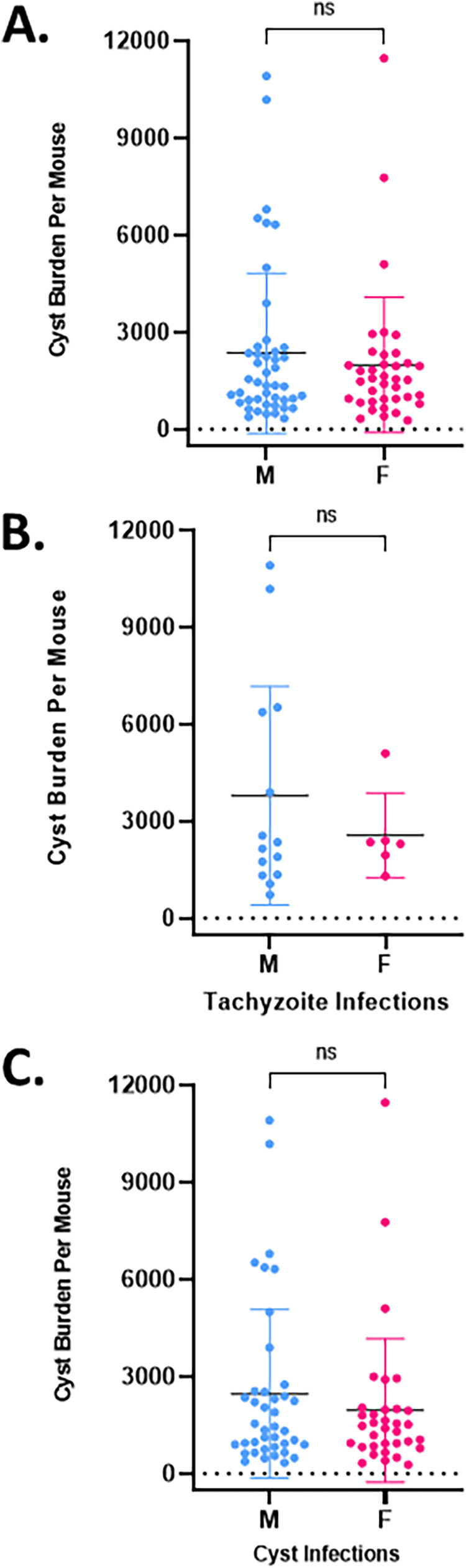
(A) Mouse sex does not impact the tissue cyst yield. Tissue cyst yields from infected male (45 preps) and female (38 preps) mice each exhibit a broad range of cyst recovery with no statistical difference based on sex. (B) Despite differences in susceptibility between female and male mice following tachyzoite infection, there is no statistical difference in the recovery of tissue cysts from surviving animals. Male, 14 preps; female, 6 preps. (C) Tissue cyst yields from male (40 preps) and female (34 preps) mice infected with tissue cysts are not statistically different. Statistical analyses using a two-tailed unpaired *t* test were repeated with log(N)-transformed data and yielded identical outcomes (Fig. S3A to 3C).

The effect of a mouse’s sex on the acute infection, its immune responses to that infection, and other aspects of the chronic infection have been investigated (25–30). Less has been done to establish whether the sex of the host animal impacts the recovery of tissue cysts for infections initiated with either tachyzoites or tissue cysts. Although sex-based differences have been described previously (25–30), w found no difference in the overall recovery of tissue cysts from male versus female mice infected with either tachyzoites (Fig. 3A and B) or tissue cysts (Fig. 3A and C). In light of the nonparametric nature of these data, statistical analysis was additionally performed on ln-transformed data sets (Fig. S3), confirming the absence of any statistical differences based on the sex of the host animal.

**FIG 4:**
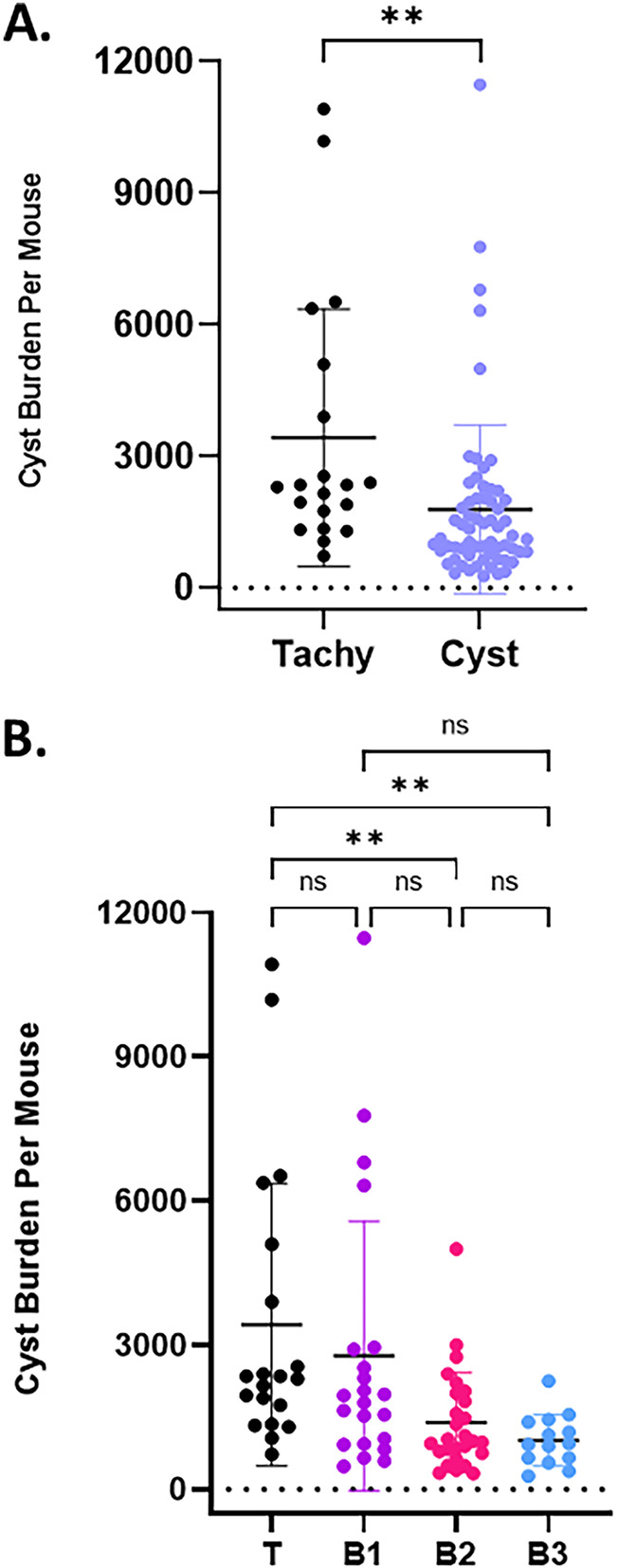
Effect of the inoculum on tissue cyst recovery. (A) Tissue cyst recovery as a function of infection with tissue culture-derived tachyzoites (Tachy: 20 preps) compared to tissue cysts (Cyst: 63 preps) reveals a significant difference in cyst recovery (two-tailed unpaired *t* test: **, *P* < 0.0050). (B) Serial passage of tissue cysts *in vivo* results in diminishing cyst yields. B1 tissue cyst (22 preps)-initiated infections yield similar tissue cyst numbers as tachyzoite (T)-initiated infections. Tissue cyst yields from B2 (27 preps)-initiated infections are statistically lower than tachyzoite-initiated infections but not lower than B1-initiated infections. B3 (14 preps) tissue cyst-initiated infections produce statistically lower yields than tachyzoite-initiated infections. Statistical analysis by one-way analysis of variance (ANOVA) revealed no statistical differences between T- and B1-initiated infections or pairwise between B1, B2, and B3. Statistical significance was noted between T and B2 (**, *P* = 0.0096) and between T and B3 (**, *P* = 0.0095). Because the data were nonparametric, log(N)-transformed data were subjected to statistical analysis (Fig. S3). Analysis of transformed data revealed both additional and more robust significance in this data set.

We next examined how the inoculum type affected the overall recovery of tissue cysts. Notably, our earlier finding that the time of harvest did not alter the cyst yield in a statistically significant manner (9) was confirmed in this study (data not shown). These results clearly indicate that tachyzoite-initiated infections consistently generate higher tissue cyst burdens than tissue cyst-initiated infections (Fig. 4A). High statistical significance was confirmed when assessed using ln-transformed data (Fig. S3). Notably, fewer tachyzoite-initiated cyst purifications met the criteria for inclusion (two animals of the same sex; see Materials and Methods) due to the high overall mortality associated with tachyzoite infections.

### Effect of serial tissue cyst passage on subsequent cyst yields.

In light of the lower mortality associated with tissue cyst-initiated infections (Fig. 1), we examined whether serial passage of tissue cysts represented a potential means to mitigate tachyzoite-associated mortality (Fig. 4B). The segregation of data based on bradyzoite passage number (B1, B2, B3) confirmed not only lower cyst recoveries compared to tachyzoite infections but also a trend of diminishing cyst yields (Fig. 4B). Notably, overall yields for B1 cysts were not statistically different for both non- (Fig. 4B) and ln-transformed (Fig. S3) data, while clear differences emerged between tachyzoite and subsequent passages (B2 and B3) (Fig. 4B, Fig. S3). Together, these results suggest that serial passage within CBA/J mice, while resulting in low overall mortality, needs to be balanced with the diminishing yields as quantified on a per-mouse infected basis (Table 1).

**TABLE 1:** Tissue cyst yields based in inoculum type^*a*^

Parameter	Inoculum type			
	T	B1	B2	B3
Total mice harvested	40	44	54	28
Total mice infected	136	67	76	40
Harvested (%)	29.4	65.7	71.1	70.0
Mean cysts per mouse harvested	3,424	2,773	1,388	1,017
Mean cysts per mouse infected	504	911	493	356

a T, tachyzoite-initiated infection; B1 to B3, bradyzoite passage number (1 to 3 passages).

### Effect of “age” at harvest on subsequent tissue cyst burden.

**FIG 5:**
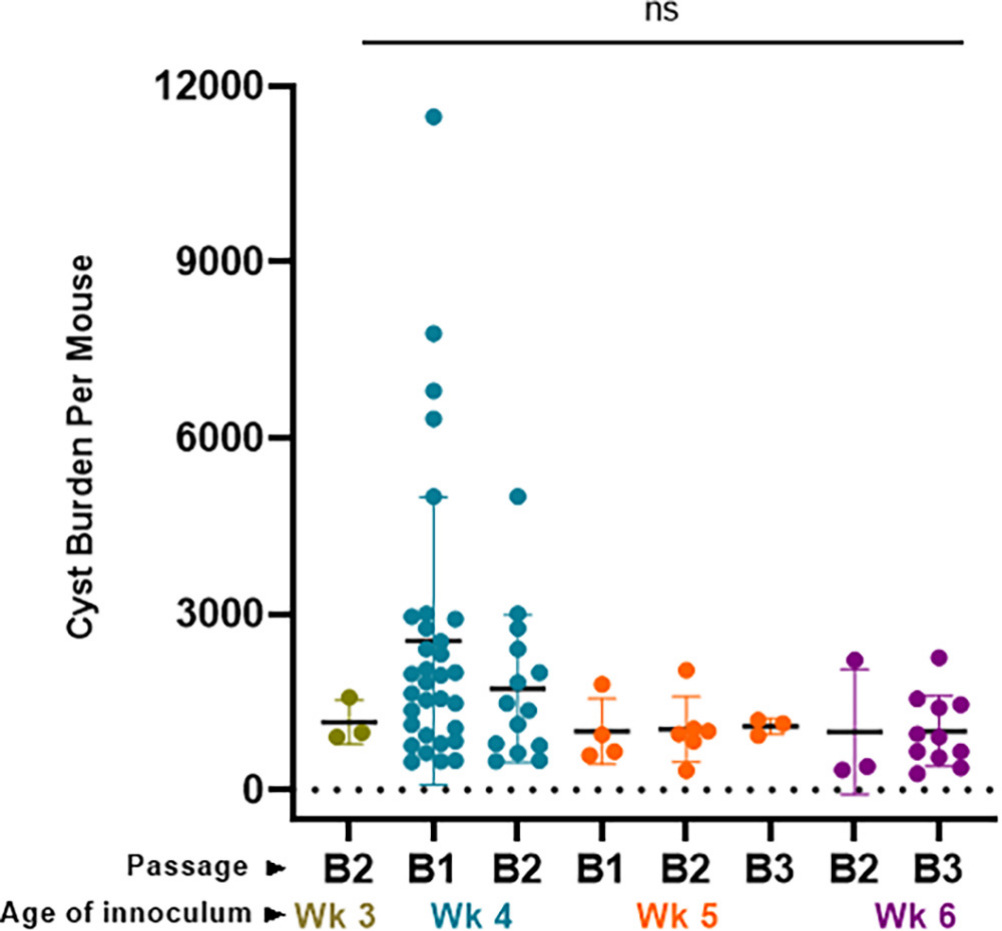
Effect of the duration of infection on subsequent infectivity. Tissue cysts harvested at 3, 4, 5, and 6 weeks postinfection, representing B1, B2, and B3 cysts, were used as the inocula to determine whether the time of harvest impacted yield in a serial passage. The age of the inoculum was found to have no significant impact on tissue cyst yield by ANOVA analysis. This finding was confirmed using log(N)-transformed data accommodating the nonparametric nature of the data set (Fig. S3). Week 3: B2, 3 preps. Week 4: B1, 32 preps; B2, 14 preps. Week 5: B1, 4 preps; B2, 6 preps; B3, 3 preps. Week 6: B2, 3 preps; B3, 11 preps.

Contrary to long-standing dogma, chronic infection is considerably more dynamic than previously thought (5, 9). TgIMC3 intensity acts as a surrogate for the birth-dating of individual bradyzoites that can serve as an indicator of active growth or relative dormancy (9). As such, the proportion of “younger,” more active bradyzoites relative to their inactive peers within the same tissue cyst can potentially impact their capacity to establish subsequent infections and thereby the overall cyst yield. We therefore reexamined the data for bradyzoite-initiated infections, resolving whether the “age” of the infection at the time of tissue cyst harvest affected the overall tissue cyst yield during the subsequent infection cycle. While it was clear that bradyzoite-initiated infections as a whole typically yielded lower cyst recovery compared to tachyzoite-initiated infections (Fig. 4A), their age at the time of harvest did not significantly impact the subsequent recovery of tissue cysts during subsequent purifications (Fig. 5A, Fig. S3). Although tissue cysts harvested at weeks 3 and 4 exhibited higher overall replicative capacity than week 5 cysts (9), their ability to form cysts in subsequent infections was not statistically significant. In addition, despite B2 and B3 cyst recovery being generally lower, their capacity to seed a fresh infection cycle following i.p. injections was not adversely impacted.

## DISCUSSION

Bradyzoites within tissue cysts are arguably the most clinically relevant form of reactivated disease upon immune suppression (10, 31, 32). Despite its central role in pathogenesis, little is known about the biology of the bradyzoite and the community of bradyzoites that represents each individual tissue cyst (5). Notably, while bradyzoites within a tissue cyst are genetically clonal, they represent a physiologically diverse population that varies from cyst to cyst even within the same animal (5, 9). As such, the bradyzoite retains a level of plasticity, permitting it to remain in this state, de-differentiate into a tachyzoite during recrudescence, or enter the sexual cycle in the feline gut (33). It is tempting to speculate that the heterogeneity inherent within the genetically clonal tissue cyst is central to the survival of the organism.

The variable effect of infection in the murine model has been well documented, with both parasite effectors and host factors engaged in a complex interplay defining susceptibility and resistance (23, 27, 32, 34–36). Beyond the inherent variability observed with cyst burdens, differences with regard to parasite lines, mouse backgrounds (23), and the sensitivity of approaches used to quantify cyst burdens present vastly different numbers, as summarized in a comprehensive review on the topic (22). These differences, together with the lack of standardization, complicate comparisons across studies, which are becoming increasingly important with the renewed focus on chronic infection. In the context of cyst formation and yield, the pairing of Type II ME49 parasites (and its derivatives, like the ΔHXGPRT line used here) and CBA/J mice is recognized as providing the optimal balance ensuring reproducibly high tissue cyst yields (22). We therefore used this specific pairing but found over the course of the studies presented here that establishing conditions for high tissue cyst yields is considerably more nuanced.

While there is no argument regarding the lethality of the mouse hypervirulent Type I strains (RH, GT1), there is considerable variation in the observed LD_50_ values dependent on the passage history of the parasite and the mouse background used in infection (22). Indeed, *in vitro* passage of ME49 in tissue culture selects for mouse hypervirulence, resulting in a reduction in the LD_50_ value of 2 orders of magnitude or greater (from 10^4^ to 100 or fewer organisms) (23, 37, 38). This was observed to be the case with the lab-adapted Type II ME49 and ME49ΔHXGPRT used here. Tachyzoite-initiated infections caused considerable mortality upon infection with 100 tachyzoites, with statistically different susceptibility profiles for male versus female animals (Fig. 1B). As such, female mice tended to get sicker earlier and succumb at higher rates relative to males in response to i.p. injection with 100 ME49 tachyzoites during acute infection (Fig. 2B). Recent work from the Knoll laboratory examining transcriptional responses within the host during the progression of chronic infection highlights clear differences in responses between sexes (38), despite the overall response profiles, based on several other criteria, being fairly similar. Notably, similar to this study, Garfoot et al. used CBA/J mice (albeit of different ages than used here) of both sexes and Type II ME49 parasites maintained in tissue culture. However, infection doses of 10^4^ tachyzoites allowed survival during the acute phase and establishment of the chronic infection (38), highlighting the importance of empirically establishing the specific tachyzoite infection regimen because sex, mouse age, and additional factors impact the outcome of the acute infection that precedes and is essential to the establishment of the chronic infection being interrogated here.

In a previous study, using exclusively female mice, we established that infection with 20 *ex vivo* tissue cysts i.p. from infected brain homogenates reproducibly generated robust cyst yields (9). Extending these findings, we confirmed that infection with tissue cysts in animals of both sexes is associated with very low mortality rates (Fig. 1C) and symptomology (Fig. 1D). Despite the absence of overt symptoms during the course of the infection, tissue cysts were recoverable from the brains of infected animals of both sexes, with no sex bias related to yield (Fig. 3). However, differences in overall yield were observed when comparing tachyzoite- versus tissue cyst-initiated infections (Fig. 4A). Of note, serial passage of tissue cysts *in vivo* resulted in a diminishing recovery, accounting for the observed differences (Fig. 4B).

Anecdotal evidence, including discussion with other investigators, prompted us to monitor additional sporadic symptomology beyond the classical disease progression (Fig. 2A). Among these symptoms, we observed the development of head tilts, ataxia in both sexes, and priapism/penile prolapse in males. The emergence of each of these conditions was first observed during the second week of infection (Fig. 2D and E), with the total number of presenting animals increasing during the late acute and early chronic stages of infection. All cases of penile prolapse/priapism were observed at the end of the acute phase and early in the chronic phase (week 3) for both tachyzoite- and cyst-initiated infections (Fig. 2D and E). Cases of ataxia with tachyzoite-initiated infections were restricted to the acute phase (Fig. 2E), while cyst-initiated infections manifested with equal onset in both the acute and chronic phases (Fig. 2D). A less robust distinction was observed for the onset of a head tilt. Here, 75% of afflicted animals presented prior to the 3-week time point with tachyzoite infections, with half (50%) of all new instances occurring prior to the 3-week point (Fig. 2D) and the remaining half presenting during the chronic phase (Fig. 2E). Instances of animals developing a head tilt for the first time later in the chronic phase are likely due to reactivation events. In addition, the lower prevalence of the onset of these symptoms later in the chronic phase in tachyzoite-initiated infections is likely due to higher mortality earlier in the course of the disease.

Examination of available literature indicates that infection-associated head tilts in mice have been noted, with bacterial infections of the inner ear (39, 40) being the primary reason for this condition. Direct brain/CNS involvement was noted in the case of a murine Plasmodium berghei study, where it may have been linked to intracerebral hemorrhages (41). Cerebral hemorrhages were also associated with increased rodent head tilts in an ischemia reperfusion injury model, independent of an infection being present (42, 43). Such CNS injuries are also linked to hemiparesis, which can also cause behaviors such as the circling observed in some animals with head tilts (Movie S1 in the supplemental material). Finally, dysregulation of inflammatory responses resulting in intracerebral inflammatory foci and linked to interferon-gamma signaling in an autoimmune encephalomyelitis model was also associated with increased incidence of head tilt (44, 45). Further indication of an association with an aberrant inflammatory response was evident in head-tilt mice encoding a specific mutation in Nox3, the NADPH oxidase involved in inflammation (46).

The contribution of CNS damage associated with motor function changes to the presentation of a head tilt and ataxia is potentially associated with the location of encystation within the brain that also coincides with the areas where reactivation is likely to cause damage. In this regard, the demonstrated distribution of *Toxoplasma* within the brain and its predilection to being located in the motor area (34, 47–49) presents a potential anatomical basis for the emergence of these symptoms. Detailed mapping of tissue cyst localization and/or evidence of recrudescence in the brains of animals exhibiting these symptoms will allow definitive correlations to be established.

The presentation of penile prolapse/priapism associated with *Toxoplasma* infection is more likely due to effects on the CNS as opposed to the male genitourinary tract (50). Priapism is noted in cases of ischemic brain injury (51, 52), associated with spinal cord injury (53). In addition, older CBA mice (not the population used in this study) have a predisposition to developing priapism (54) which could potentially be exacerbated in the context of the altered neuroinflammatory environment of the infected brain.

The recovery of tissue cysts following infection with either tachyzoites or encysted bradyzoites exhibited no sex-specific differences (Fig. 3), suggesting that the differences seen following tachyzoite versus cyst infection (Fig. 4) were likely due to differences in the host response as opposed to factors intrinsic to the parasite. The more aggressive host response in tachyzoites paradoxically resulted in significantly higher tissue cyst recovery in surviving mice compared to cumulative recovery following cyst infection, where lower symptomology and mortality was observed (Fig. 4A). This finding suggests that maintenance of the infection by serial passage of brain-derived *ex vivo* tissue cysts would effectively circumvent the issue of tachyzoite infection-associated mortality which precludes cyst recovery. Unfortunately, our data establish that serial passage of tissue cysts results in a progressive trend toward reduced overall recovery of cysts (Fig. 4B). This trend of decreasing cyst yields was not observed in a ME49 line maintained in the Wilson laboratory by serial passage over multiple years in mice (37). One key difference in this maintenance protocol is the “recharging” of that line in outbred Swiss Webster animals (37), where presumed epigenetic changes are reinstituted to permit the restoration of high cyst yields upon return to infections in inbred mice.

Our prior and ongoing analysis of bradyzoite replication within tissue cysts points to largely asynchronous growth that broadly follows a cyclical pattern, at least during the first 8 weeks of infection (9). The inner membrane complex protein TgIMC3 (55) serves as a reporter for both parasite birth-dating as well as determining their relative age based on the intensity of labeling (9). Thus, early in the chronic phase, at week 3 postinfection (TgIMC3 high), parasites are in a higher state of metabolic activity associated with extensive replication, both recent and active (9). At week 5 postinfection, this activity is universally low (9), recovering to an intermediate level by week 8 (9). We therefore reasoned that infection of mice with more “active” cysts could result in overall higher cyst yields. However, this was not found to be the case (Fig. 5). Rather, for bradyzoite-initiated infections, the passage cycle (B1, B2, B3) (Fig. 4 and 5) appeared to be a primary determinant of subsequent cyst recovery, suggesting that the apparent reprogramming by continuous maintenance in a given mouse background (CBA/J in this case) is a key determinant for tissue cyst-initiated infections.

On a practical level, our findings reveal that the design of studies in which tissue cyst yield is important need to consider the source of the inoculum (tachyzoite versus bradyzoite [B1, B2, B3]), where attrition during the acute phase is balanced by subsequent yield (Table 1). With this consideration, B1 tissue cysts derived from tachyzoite-initiated infections provide optimal recovery per infected mouse (Table 1). As we seek insights into the physiology of *in vivo*-sourced encysted bradyzoites, optimization of recovery is likely to play an important role.

Insights into bradyzoite physiology are primarily derived from *in vitro* studies using stress-induced stage conversion (56–59). These studies, while important for addressing the machinery of stage conversion, do not provide meaningful insights into the actual biology of bradyzoites *in vivo*. What limited studies reveal is that despite being genetically clonal, individual tissue cysts exhibit considerable physiological heterogeneity with regard to the resident bradyzoites (5, 9). Our development of imaging-based approaches to quantify bradyzoite numbers within *ex vivo* purified cysts revealed the scope of this heterogeneity (5, 9), a feature that is reinforced by ongoing studies with the development of imaging-based quantitative physiological readouts, including quantification of mitochondrial morphology and activity (60), amylopectin, and replication potential (Patwardhan and Sinai, unpublished data). These tools are revealing insights into bradyzoite physiology that will inform the discovery and development of drugs against this currently intractable life cycle stage.

One fundamental limitation in the evaluation of potential drugs effective during chronic infection is the fact that efficacy, measured by the apparent reduction in cyst burdens following treatment, is presented in terms of percentage (10, 12, 13, 15, 16, 61). While this metric of sensitivity is the currently acceptable norm, it fails to address the mechanistic basis for drug susceptibility or resistance. Initiating investigation at the level of resident bradyzoites with an appreciation for their heterogeneity will lead to more meaningful insights into the biology of this recalcitrant life cycle stage.

Our findings that variations in cyst yield are analogous to what is reported for drug treatment (Fig. 3,4) are influenced by nature of the initial inoculum, passage, and other not-yet determined factors highlight the inefficiency and potential inaccuracy of using tissue cyst recovery as the sole metric of drug effectiveness. These issues can be compounded further when using solely the cyst burden to assign functional attributes to mutations in mutant parasite lines (59, 62, 63). As with studies on drug efficacy, the experimental design has to be powerful enough to account for the inherent variability in cyst recovery with wild-type or parental parasites. As the field continues to recognize that tissue cysts and the bradyzoites they house are not dormant entities, directed physiologic, metabolomic, and transcriptomic studies which depend on the ability to recover high cyst numbers for analysis will become increasingly critical. Our objective in compiling these study data is to highlight the factors that drive cyst yield, all of which are subject to further refinement. We hope this work will encourage further studies in the field aimed at optimizing the recovery of tissue cysts to promote meaningful in-depth studies into this critical and fascinating life cycle stage of this parasite.

## MATERIALS AND METHODS

### Parasite line.

All infections using both tachyzoites and *ex vivo* brain-derived tissue cysts utilized a derivative of Type II ME49 parasites with a deletion of the HXGPRT (TGME49_200320) gene (17). This line was generated using CRISPR-Cas9-mediated deletion of the gene (20, 21) followed by selection with 6TX (17) (Fig. S1A and B). Selection of the targeting single guide RNA sequences followed published sequences targeting this gene (18). Specifically, TgHXGRPT (Exon2, positive strand), ATGGTCTCCACCAGTGCTCC; TgHXGPRT (Exon3, negative strand), GACAAAATCCTCCTCCCTGG; and TgHXGPRT (Exon5, positive strand), CTTCTTCGAGCACTTGTCC were introduced into the pSAG1::CAS(-GFP::sgUPRT) plasmid (20, 21) to generate 3 distinct mutagenesis plasmids that were co-transfected into Type II ME49 parasites. Initial enrichment for transfected parasites was achieved using flow cytometry (64) prior to selection with 6-TX at 80 mg/mL in minimal essential medium α (MEMα) with 7% dialyzed fetal bovine serum (FBS). Following drug selection, cloning, and confirmation of the mutation (Fig. S1C), parasites were maintained by serial passage in human foreskin fibroblast (HFF) cells grown in bicarbonate-buffered MEMα supplemented with 7% heat-inactivated FBS, 50 mM glutamine, and 25 mM penicillin/streptomycin at 37°C in 5% CO_2_. This derivative exhibited identical growth characteristics (Fig. S1D) and *in vitro* switching in response to pH 8.2 ambient CO_2_ incubation compared to wild-type ME49 parasites obtained from the AIDS Resource Center (Fig. S1E).

### Establishment of growth characteristics and *in vitro* switching for ME49ΔHX line.

The growth characteristics of the ME49ΔHX line were established using a conventional plaque assay. Confluent primary HFF cells were established in 12-well plates under standard cell culture conditions. Monolaters were infected with 200 tachyzoites per well and incubated undisturbed for 6 days. The infected monolayers were fixed with −20°C MeOH for 20 min and stained with 1% crystal violet for 20 min with gentle rocking, followed by de-staining with multiple washes in tap water. Plaques were imaged using a flatbed photographic scanner at 600 dpi and the pixel area for clearing was measured using ImageJ. Data from three independent replicates were compiled.

*In vitro* switching under alkaline stress (pH 8.2, ambient CO_2_, 37°C) conditions was evaluated at days 2, 4, and 6 postinfection of HFF cells based on the detection of cyst wall formation using FITC (fluorescein isothiocyanate)-conjugated *Dolichos biflorus* lectin agglutinin (DBA; Vector Laboratories). Images of developing tissue cysts were acquired at random at the specific time points on blinded samples using fixed exposure. DBA intensity was measured using Image J on 8-bit grayscale images. Data were pooled from 3 independent experiments.

### Mouse infection model.

Both male and female CBA/J mice (strain no. 00065, Jackson Laboratory, Bar Harbor, ME) were used in these studies. Mice were procured at 4 to 6 weeks and habituated in the animal facility for a minimum of 1 week, provided with standard chow (Teklad 2918 irradiated 18% CP rodent diet, Envigo, Indianapolis, IN) and water *ad libitum* during the course of these studies. Mice were infected with *Toxoplasma* by i.p. injection typically within 2.5 weeks of receipt from the vendor. Infection with cell culture-derived tachyzoites was performed using syringe-passaged parasites recovered in phosphate-buffered saline and kept on ice. At least two independent counts were used to establish the number of tachyzoites in the seed stock. Inocula containing 100 tachyzoites/200 μL per mouse (500 tachyzoites/mL) were diluted into serum-free Opti-MEM (Gibco). The inoculating suspension was maintained on ice and 200 μL was injected i.p. into each mouse. Prior to injection, mice were mildly anesthetized with 30% isoflurane in propylene glycol using a drop jar method (65), as required by the Institutional Animal Care and Use Committee (IACUC) for injection of infectious agents.

Infection of animals with brain-derived tissue cysts was performed by diluting a reserved volume of brain homogenate obtained during purification of brain-derived tissue cysts. Brain homogenate corresponding to 20 cysts diluted to 200 μL using Opti-MEM was used as the inoculum. Quantification of tissue cyst burdens within brain homogenates was established as previously described below (14).

In this study, in addition to tracking the tissue cyst burden from tachyzoite-initiated (T) infection, we monitored the effect of serial passage of tissue cysts on overall tissue cyst burdens. Accordingly, the first cycle of infection initiated from tissue cysts (bradyzoites) generated from a tachyzoite-initiated infection represented the B1 inoculum. Subsequent serial passages of tissue cysts from B1 initiated infections were designated B2, followed by B3 (Fig. 1A).

Following infection, mice were monitored daily for the development of symptoms, with monitoring increased to at least twice daily once animals became symptomatic. The progression of infection was monitored using a body score index rubric (Fig. 2A). Notably, this rubric, which reflects the typical progression of symptoms, does not always follow the exact progression, but it does provide a framework to standardize symptom severity based on physical observation. As symptoms progressed, supportive treatment was limited to the provision of a gel-based diet (DietGel 76A, Clear H_2_O, Westbrook, ME) and wet chow on the cage floor. Subdermal saline injections were administered to animals which presented as dehydrated. No additional interventions were provided to symptomatic animals because the administration of either anti-*Toxoplasma* treatments or anti-inflammatory drugs would alter the progression to the encysted state. All studies involving the use of animals and biohazards were approved by the IACUC and Institutional Biosafety Committees (IBC), respectively, at the University of Kentucky.

### Tissue cyst purification and quantification.

Tissue cyst purifications were performed using a modified Percoll gradient method (9, 14) based on the classic Cornelison protocol (66). Of note, all tissue cyst yields reported in this work were generated from Percoll gradients loaded with the brain homogenates from the brains of two animals of the same sex. Although mixed-sex gradients were run, these data are excluded from the current analysis. Additionally, tissue cysts recovered from running single-brain gradients using a modified protocol (14) are not reported here because prior work has demonstrated that recovery of cysts from two brain gradients is optimal, with significantly lower yields obtained from single brain gradients (14). Quantification of cyst burdens was established by summation of the relative cyst numbers detected in Percoll fractions as previously described (9, 14). Mean yield per mouse was calculated from the total yield of a Percoll gradient, resulting in a single data point from a two-brain preparation.

### Statistical analysis.

Mouse mortality for each sex following either tachyzoite- or tissue cyst-initiated infection was evaluated using a Kaplan-Meier estimator. Additionally, a graphical representation of disease progression based on body score index data rubric over the first 28 days of infection was plotted as a stacked graph.

The distribution of data related to tissue cyst yield accumulated over the course of this study was found to be nonparametric. We therefore performed statistical analyses with both the raw data (figures) and following a log(N) transformation (ln-transform) of the data (Fig. S3). All statistical analyses were performed using GraphPad Prism, with the specific tests used indicated in the figure legends.

## References

[1] Dubey JP, Jones JL. 2008. *Toxoplasma gondii* infection in humans and animals in the United States. Int J Parasitol 38:1257–1278. doi:10.1016/j.ijpara.2008.03.007. 18508057

[2] Wang ZD, Wang SC, Liu HH, Ma HY, Li ZY, Wei F, Zhu XQ, Liu Q. 2017. Prevalence and burden of *Toxoplasma gondii* infection in HIV-infected people: a systematic review and meta-analysis. Lancet HIV 4:e177–e188. doi:10.1016/S2352-3018(17)30005-X. 28159548

[3] Alvarez Garcia G, Davidson R, Jokelainen P, Klevar S, Spano F, Seeber F. 2021. Identification of oocyst-driven *Toxoplasma gondii* infections in humans and animals through stage-specific serology-current status and future perspectives. Microorganisms 9:2346. doi:10.3390/microorganisms9112346. 34835471 PMC8618849

[4] Zhu S, Shapiro K, VanWormer E. 2022. Dynamics and epidemiology of *Toxoplasma gondii* oocyst shedding in domestic and wild felids. Transbound Emerg Dis 69:2412–2423. doi:10.1111/tbed.14197. 34153160

[5] Sinai AP, Watts EA, Dhara A, Murphy RD, Gentry MS, Patwardhan A. 2016. Reexamining chronic *Toxoplasma gondii* infection: surprising activity for a “dormant” parasite. Curr Clin Microbiol Rep 3:175–185. doi:10.1007/s40588-016-0045-3. 28191447 PMC5295825

[6] Dubey JP. 2009. History of the discovery of the life cycle of *Toxoplasma gondii*. Int J Parasitol 39:877–882. doi:10.1016/j.ijpara.2009.01.005. 19630138

[7] Jones JL, Dubey JP. 2012. Foodborne toxoplasmosis. Clin Infect Dis 55:845–851. doi:10.1093/cid/cis508. 22618566

[8] Scallan E, Hoekstra RM, Mahon BE, Jones TF, Griffin PM. 2015. An assessment of the human health impact of seven leading foodborne pathogens in the United States using disability adjusted life years. Epidemiol Infect 143:2795–2804. doi:10.1017/S0950268814003185. 25633631 PMC9151020

[9] Watts E, Zhao Y, Dhara A, Eller B, Patwardhan A, Sinai AP. 2015. Novel approaches reveal that *Toxoplasma gondii* bradyzoites within tissue cysts are dynamic and replicating entities *in vivo*. mBio 6:e01155-15. doi:10.1128/mBio.01155-15. 26350965 PMC4600105

[10] Alday PH, Doggett JS. 2017. Drugs in development for toxoplasmosis: advances, challenges, and current status. Drug Des Devel Ther 11:273–293. doi:10.2147/DDDT.S60973. PMC5279849 28182168

[11] Doggett JS, Nilsen A, Forquer I, Wegmann KW, Jones-Brando L, Yolken RH, Bordon C, Charman SA, Katneni K, Schultz T, Burrows JN, Hinrichs DJ, Meunier B, Carruthers VB, Riscoe MK. 2012. Endochin-like quinolones are highly efficacious against acute and latent experimental toxoplasmosis. Proc Natl Acad Sci USA 109:15936–15941. doi:10.1073/pnas.1208069109. 23019377 PMC3465437

[12] McPhillie M, Zhou Y, El Bissati K, Dubey J, Lorenzi H, Capper M, Lukens AK, Hickman M, Muench S, Verma SK, Weber CR, Wheeler K, Gordon J, Sanders J, Moulton H, Wang K, Kim TK, He Y, Santos T, Woods S, Lee P, Donkin D, Kim E, Fraczek L, Lykins J, Esaa F, Alibana-Clouser F, Dovgin S, Weiss L, Brasseur G, Wirth D, Kent M, Hood L, Meunieur B, Roberts CW, Hasnain SS, Antonyuk SV, Fishwick C, McLeod R. 2016. New paradigms for understanding and step changes in treating active and chronic, persistent apicomplexan infections. Sci Rep 6:29179. doi:10.1038/srep29179. 27412848 PMC4944145

[13] McPhillie MJ, Zhou Y, Hickman MR, Gordon JA, Weber CR, Li Q, Lee PJ, Amporndanai K, Johnson RM, Darby H, Woods S, Li ZH, Priestley RS, Ristroph KD, Biering SB, El Bissati K, Hwang S, Hakim FE, Dovgin SM, Lykins JD, Roberts L, Hargrave K, Cong H, Sinai AP, Muench SP, Dubey JP, Prud'homme RK, Lorenzi HA, Biagini GA, Moreno SN, Roberts CW, Antonyuk SV, Fishwick CWG, McLeod R. 2020. Potent tetrahydroquinolone eliminates apicomplexan parasites. Front Cell Infect Microbiol 10:203. doi:10.3389/fcimb.2020.00203. 32626661 PMC7311950

[14] Watts EA, Dhara A, Sinai AP. 2017. Purification *Toxoplasma gondii* tissue cysts using Percoll gradients. Curr Protoc Microbiol 45:20C 2 1–20C 2 19. doi:10.1002/cpmc.30. PMC5568674 28510363

[15] Martynowicz J, Doggett JS, Sullivan WJ Jr. 2020. Efficacy of Guanabenz combination therapy against chronic toxoplasmosis across multiple mouse strains. Antimicrob Agents Chemother 64:e00539-20. doi:10.1128/AAC.00539-20. 32540979 PMC7449173

[16] Vidadala RS, Rivas KL, Ojo KK, Hulverson MA, Zambriski JA, Bruzual I, Schultz TL, Huang W, Zhang Z, Scheele S, DeRocher AE, Choi R, Barrett LK, Siddaramaiah LK, Hol WG, Fan E, Merritt EA, Parsons M, Freiberg G, Marsh K, Kempf DJ, Carruthers VB, Isoherranen N, Doggett JS, Van Voorhis WC, Maly DJ. 2016. Development of an orally available and central nervous system (CNS) penetrant *Toxoplasma gondii* calcium-dependent protein kinase 1 (TgCDPK1) inhibitor with minimal human ether-a-go-go-related gene (hERG) activity for the treatment of toxoplasmosis. J Med Chem 59:6531–6546. doi:10.1021/acs.jmedchem.6b00760. 27309760 PMC5100899

[17] Donald RG, Roos DS. 1998. Gene knock-outs and allelic replacements in *Toxoplasma gondii*: HXGPRT as a selectable marker for hit-and-run mutagenesis. Mol Biochem Parasitol 91:295–305. doi:10.1016/s0166-6851(97)00210-7. 9566522

[18] Sidik SM, Huet D, Ganesan SM, Huynh MH, Wang T, Nasamu AS, Thiru P, Saeij JP, Carruthers VB, Niles JC, Lourido S. 2016. A genome-wide CRISPR screen in *Toxoplasma* identifies essential apicomplexan genes. Cell 166:1423–1435.e12. doi:10.1016/j.cell.2016.08.019. 27594426 PMC5017925

[19] Donald RG, Carter D, Ullman B, Roos DS. 1996. Insertional tagging, cloning, and expression of the *Toxoplasma gondii* hypoxanthine-xanthine-guanine phosphoribosyltransferase gene. Use as a selectable marker for stable transformation. J Biol Chem 271:14010–14019. doi:10.1074/jbc.271.24.14010. 8662859

[20] Shen B, Brown K, Long S, Sibley LD. 2017. Development of CRISPR/Cas9 for efficient genome editing in *Toxoplasma gondii*. Methods Mol Biol 1498:79–103. doi:10.1007/978-1-4939-6472-7_6. 27709570

[21] Shen B, Brown KM, Lee TD, Sibley LD. 2014. Efficient gene disruption in diverse strains of *Toxoplasma gondii* using CRISPR/CAS9. mBio 5:e01114-14. doi:10.1128/mBio.01114-14. 24825012 PMC4030483

[22] Watson GF, Davis PH. 2019. Systematic review and meta-analysis of variation in *Toxoplasma gondii* cyst burden in the murine model. Exp Parasitol 196:55–62. doi:10.1016/j.exppara.2018.12.003. 30562481 PMC6447088

[23] Dubey JP. 1998. Comparative infectivity of *Toxoplasma gondii* bradyzoites in rats and mice. J Parasitol 84:1279–1282. doi:10.2307/3284691. 9920331

[24] Glausen TG, Carrillo GL, Jin RM, Boyle JP, Saeij JPJ, Wohlfert EA, Fox MA, Blader IJ. 2021. The *Toxoplasma* polymorphic effector GRA15 mediates seizure induction by modulating interleukin-1 signaling in the brain. mBio 12:e0133121. doi:10.1128/mBio.01331-21. 34154412 PMC8262954

[25] Alonaizan R, Woods S, Hargrave KE, Roberts CW. 2021. An exaggerated immune response in female BALB/c mice controls initial *Toxoplasma gondii* multiplication but increases mortality and morbidity relative to male mice. Pathogens 10:1154. doi:10.3390/pathogens10091154. 34578186 PMC8470933

[26] Gatkowska J, Wieczorek M, Dziadek B, Dzitko K, Dlugonska H. 2013. Sex-dependent neurotransmitter level changes in brains of *Toxoplasma gondii* infected mice. Exp Parasitol 133:1–7. doi:10.1016/j.exppara.2012.10.005. 23098668

[27] Kaňková S, Holáň V, Zajícová A, Kodym P, Flegr J. 2010. Modulation of immunity in mice with latent toxoplasmosis: the experimental support for the immunosuppression hypothesis of Toxoplasma-induced changes in reproduction of mice and humans. Parasitol Res 107:1421–1427. doi:10.1007/s00436-010-2013-9. 20721578

[28] Li YE, Kannan G, Pletnikov MV, Yolken RH, Xiao J. 2015. Chronic infection of *Toxoplasma gondii* downregulates miR-132 expression in multiple brain regions in a sex-dependent manner. Parasitology 142:623–632. doi:10.1017/S003118201400167X. 25351997 PMC4428143

[29] Roberts CW, Cruickshank SM, Alexander J. 1995. Sex-determined resistance to *Toxoplasma gondii* is associated with temporal differences in cytokine production. Infect Immun 63:2549–2555. doi:10.1128/iai.63.7.2549-2555.1995. 7790068 PMC173341

[30] Xiao J, Kannan G, Jones-Brando L, Brannock C, Krasnova IN, Cadet JL, Pletnikov M, Yolken RH. 2012. Sex-specific changes in gene expression and behavior induced by chronic *Toxoplasma* infection in mice. Neuroscience 206:39–48. doi:10.1016/j.neuroscience.2011.12.051. 22240252

[31] Pereira-Chioccola VL, Vidal JE, Su C. 2009. *Toxoplasma gondii* infection and cerebral toxoplasmosis in HIV-infected patients. Future Microbiol 4:1363–1379. doi:10.2217/fmb.09.89. 19995194

[32] Vidal JE. 2019. HIV-related cerebral toxoplasmosis revisited: current concepts and controversies of an old disease. J Int Assoc Provid AIDS Care 18:2325958219867315. doi:10.1177/2325958219867315. 31429353 PMC6900575

[33] Sinai AP, Suvorova ES. 2020. The RESTRICTION checkpoint: a window of opportunity governing developmental transitions in *Toxoplasma gondii*. Curr Opin Microbiol 58:99–105. doi:10.1016/j.mib.2020.09.009. 33065371 PMC8019522

[34] Berenreiterová M, Flegr J, Kuběna AA, Němec P. 2011. The distribution of *Toxoplasma gondii* cysts in the brain of a mouse with latent toxoplasmosis: implications for the behavioral manipulation hypothesis. PLoS One 6:e28925. doi:10.1371/journal.pone.0028925. 22194951 PMC3237564

[35] Brinkmann V, Remington JS, Sharma SD. 1987. Protective immunity in toxoplasmosis: correlation between antibody response, brain cyst formation, T-cell activation, and survival in normal and B-cell-deficient mice bearing the H-2k haplotype. Infect Immun 55:990–994. doi:10.1128/iai.55.4.990-994.1987. 3493977 PMC260450

[36] Saeij JP, Boyle JP, Coller S, Taylor S, Sibley LD, Brooke-Powell ET, Ajioka JW, Boothroyd JC. 2006. Polymorphic secreted kinases are key virulence factors in toxoplasmosis. Science 314:1780–1783. doi:10.1126/science.1133690. 17170306 PMC2646183

[37] Goerner AL, Vizcarra EA, Hong DD, Bergersen KV, Alvarez CA, Talavera MA, Wilson EH, White MW. 2020. An *ex vivo* model of *Toxoplasma* recrudescence. bioRxiv. doi:10.1101/2020.05.18.101931. PMC10653814 37675999

[38] Garfoot AL, Cervantes PW, Knoll LJ. 2019. Transcriptional analysis shows a robust host response to *Toxoplasma gondii* during early and late chronic infection in both male and female mice. Infect Immun 87:e00024-19. doi:10.1128/IAI.00024-19. 30858341 PMC6479041

[39] Floyd R, Michel AO, Piersigilli A, Aronowitz E, Voss HU, Ricart Arbona RJ. 2021. Ethmoidal meningoencephalocele in a C57BL/6J mouse. Lab Anim 55:181–188. doi:10.1177/0023677220944449. 32787540 PMC8404781

[40] Collymore C, Giuliano F, Banks EK. 2019. Head tilt in immunodeficient mice due to contamination of drinking water by *Burkholderia gladioli*. J Am Assoc Lab Anim Sci 58:246–250. doi:10.30802/AALAS-JAALAS-18-000106. 30764891 PMC6433346

[41] Recuenco FC, Takano R, Chiba S, Sugi T, Takemae H, Murakoshi F, Ishiwa A, Inomata A, Horimoto T, Kobayashi Y, Horiuchi N, Kato K. 2014. Lambda-carrageenan treatment exacerbates the severity of cerebral malaria caused by *Plasmodium berghei* ANKA in BALB/c mice. Malar J 13:487. doi:10.1186/1475-2875-13-487. 25495520 PMC4295290

[42] Southard T, Brayton CF. 2011. Spontaneous unilateral brainstem infarction in Swiss mice. Vet Pathol 48:726–729. doi:10.1177/0300985810370155. 20466861

[43] Van der Donckt C, Van Herck JL, Schrijvers DM, Vanhoutte G, Verhoye M, Blockx I, Van Der Linden A, Bauters D, Lijnen HR, Sluimer JC, Roth L, Van Hove CE, Fransen P, Knaapen MW, Hervent AS, De Keulenaer GW, Bult H, Martinet W, Herman AG, De Meyer GR. 2015. Elastin fragmentation in atherosclerotic mice leads to intraplaque neovascularization, plaque rupture, myocardial infarction, stroke, and sudden death. Eur Heart J 36:1049–1058. doi:10.1093/eurheartj/ehu041. 24553721 PMC4416138

[44] Lee E, Chanamara S, Pleasure D, Soulika AM. 2012. IFN-gamma signaling in the central nervous system controls the course of experimental autoimmune encephalomyelitis independently of the localization and composition of inflammatory foci. J Neuroinflammation 9:7. doi:10.1186/1742-2094-9-7. 22248039 PMC3293042

[45] Wensky AK, Furtado GC, Marcondes MC, Chen S, Manfra D, Lira SA, Zagzag D, Lafaille JJ. 2005. IFN-gamma determines distinct clinical outcomes in autoimmune encephalomyelitis. J Immunol 174:1416–1423. doi:10.4049/jimmunol.174.3.1416. 15661899

[46] Paffenholz R, Bergstrom RA, Pasutto F, Wabnitz P, Munroe RJ, Jagla W, Heinzmann U, Marquardt A, Bareiss A, Laufs J, Russ A, Stumm G, Schimenti JC, Bergstrom DE. 2004. Vestibular defects in head-tilt mice result from mutations in Nox3, encoding an NADPH oxidase. Genes Dev 18:486–491. doi:10.1101/gad.1172504. 15014044 PMC374230

[47] Mendez OA, Flores Machado E, Lu J, Koshy AA. 2021. Injection with *Toxoplasma gondii* protein affects neuron health and survival. Elife 10:67681. doi:10.7554/eLife.67681. PMC8270641 34106047

[48] Boillat M, Hammoudi PM, Dogga SK, Pages S, Goubran M, Rodriguez I, Soldati-Favre D. 2020. Neuroinflammation-associated aspecific manipulation of mouse predator fear by *Toxoplasma gondii*. Cell Rep 30:320–334 e6. doi:10.1016/j.celrep.2019.12.019. 31940479 PMC6963786

[49] Dellacasa-Lindberg I, Hitziger N, Barragan A. 2007. Localized recrudescence of *Toxoplasma* infections in the central nervous system of immunocompromised mice assessed by *in vivo* bioluminescence imaging. Microbes Infect 9:1291–1298. doi:10.1016/j.micinf.2007.06.003. 17897859

[50] Colinot DL, Garbuz T, Bosland MC, Wang L, Rice SE, Sullivan WJ Jr, Arrizabalaga G, Jerde TJ. 2017. The common parasite *Toxoplasma gondii* induces prostatic inflammation and microglandular hyperplasia in a mouse model. Prostate 77:1066–1075. doi:10.1002/pros.23362. 28497488 PMC6826344

[51] Akhoundzadeh K, Vakili A. 2019. Occurrence of priapism after transient right MCAO in Swiss albino mice. Somatosens Mot Res 36:151–155. doi:10.1080/08990220.2019.1632182. 31230504

[52] Tang Y, Yuan F, Cai B, Xia W, Wang Y, Yang GY. 2016. Effect of ischaemic brain injury on sexual function in adult mice. Stroke Vasc Neurol 1:127–132. doi:10.1136/svn-2016-000013. 28959474 PMC5435199

[53] Allard J, Edmunds NJ. 2008. Reflex penile erection in anesthetized mice: an exploratory study. Neuroscience 155:283–290. doi:10.1016/j.neuroscience.2008.05.027. 18599219

[54] Adams DD, Adams JD, Lucas WO, Springford JS, Berkeley BB. 1993. A monogenic senility syndrome segregating with longevity in mice. Mechanisms of Ageing and Development 67:269–287. doi:10.1016/0047-6374(93)90005-c. 8326748

[55] Anderson-White BR, Ivey FD, Cheng K, Szatanek T, Lorestani A, Beckers CJ, Ferguson DJ, Sahoo N, Gubbels MJ. 2011. A family of intermediate filament-like proteins is sequentially assembled into the cytoskeleton of *Toxoplasma gondii*. Cell Microbiol 13:18–31. doi:10.1111/j.1462-5822.2010.01514.x. 20698859 PMC3005026

[56] Sullivan WJ Jr, Jeffers V. 2012. Mechanisms of *Toxoplasma gondii* persistence and latency. FEMS Microbiol Rev 36:717–733. doi:10.1111/j.1574-6976.2011.00305.x. 22091606 PMC3319474

[57] Krishnan A, Kloehn J, Lunghi M, Chiappino-Pepe A, Waldman BS, Nicolas D, Varesio E, Hehl A, Lourido S, Hatzimanikatis V, Soldati-Favre D. 2020. Functional and computational genomics reveal unprecedented flexibility in stage-specific *Toxoplasma* metabolism. Cell Host Microbe 27:290–306 e11. doi:10.1016/j.chom.2020.01.002. 31991093

[58] Bohne W, Heesemann J, Gross U. 1994. Reduced replication of *Toxoplasma gondii* is necessary for induction of bradyzoite-specific antigens: a possible role for nitric oxide in triggering stage conversion. Infect Immun 62:1761–1767. doi:10.1128/iai.62.5.1761-1767.1994. 8168938 PMC186404

[59] Waldman BS, Schwarz D, Wadsworth MH 2nd, Saeij JP, Shalek AK, Lourido S. 2020. Identification of a master regulator of differentiation in *Toxoplasma*. Cell 180:359–372 e16. doi:10.1016/j.cell.2019.12.013. 31955846 PMC6978799

[60] Place BC, Troublefield CA, Murphy RD, Sinai AP, Patwardhan AR. 2023. Machine learning based classification of mitochondrial morphologies from fluorescence microscopy images of *Toxoplasma gondii* cysts. PLoS One 18:e0280746. doi:10.1371/journal.pone.0280746. 36730225 PMC9894464

[61] Doggett JS, Schultz T, Miller AJ, Bruzual I, Pou S, Winter R, Dodean R, Zakharov LN, Nilsen A, Riscoe MK, Carruthers VB. 2020. Orally bioavailable endochin-like quinolone carbonate ester prodrug reduces *Toxoplasma gondii* brain cysts. Antimicrob Agents Chemother 64:e00535-20. doi:10.1128/AAC.00535-20. 32540978 PMC7449172

[62] Uboldi AD, McCoy JM, Blume M, Gerlic M, Ferguson DJ, Dagley LF, Beahan CT, Stapleton DI, Gooley PR, Bacic A, Masters SL, Webb AI, McConville MJ, Tonkin CJ. 2015. Regulation of starch stores by a Ca^2+^-dependent protein kinase is essential for viable cyst development in *Toxoplasma gondii*. Cell Host Microbe 18:670–681. doi:10.1016/j.chom.2015.11.004. 26651943

[63] Radke JB, Lucas O, De Silva EK, Ma Y, Sullivan WJ Jr, Weiss LM, Llinas M, White MW. 2013. ApiAP2 transcription factor restricts development of the *Toxoplasma tissue* cyst. Proc Natl Acad Sci USA 110:6871–6876. doi:10.1073/pnas.1300059110. 23572590 PMC3637731

[64] Dhara A, de Paula Baptista R, Kissinger JC, Snow EC, Sinai AP. 2017. Ablation of an ovarian tumor family deubiquitinase exposes the underlying regulation governing the plasticity of cell cycle progression in *Toxoplasma gondii*. mBio 8:e01846-17. doi:10.1128/mBio.01846-17. 29162714 PMC5698556

[65] Nagate T, Chino T, Nishiyama C, Okuhara D, Tahara T, Maruyama Y, Kasahara H, Takashima K, Kobayashi S, Motokawa Y, Muto S, Kuroda J. 2007. Diluted isoflurane as a suitable alternative for diethyl ether for rat anaesthesia in regular toxicology studies. J Vet Med Sci 69:1137–1143. doi:10.1292/jvms.69.1137. 18057828

[66] Cornelissen AW, Overdulve JP, Hoenderboom JM. 1981. Separation of *Isospora* (*Toxoplasma*) *gondii* cysts and cystozoites from mouse brain tissue by continuous density-gradient centrifugation. Parasitology 83:103–108. doi:10.1017/s0031182000050071. 6267543

