# Supplementary figures and images for: Factors Influencing Tissue Cyst Yield in a Murine Model of Chronic Toxoplasmosis
Source: Infect Immun. 2023 Jun 26;91(7):e00566-22. doi: 10.1128/iai.00566-22 (PMC10353391; doi:10.1128/iai.00566-22)

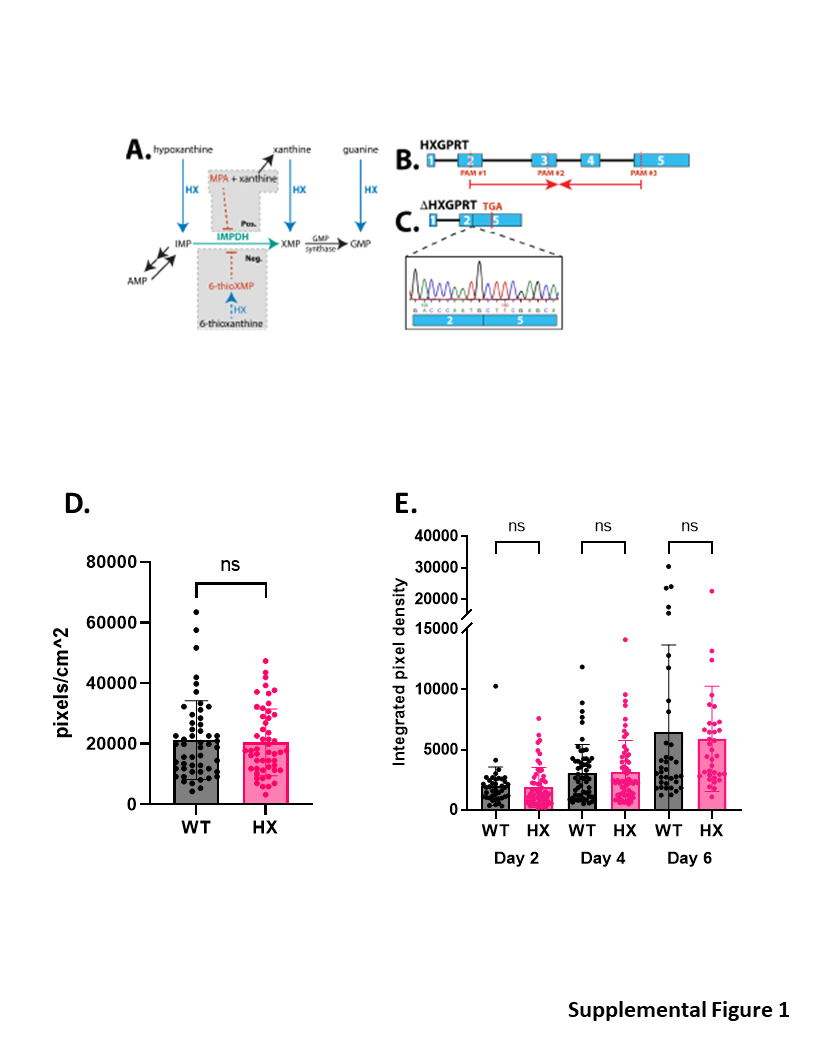

Supplement: Supplemental file 1 — Fig. S1. Download iai.00566-22-s0002.tif, TIF file, 0.1 MB [file iai.00566-22-s0002.tif]

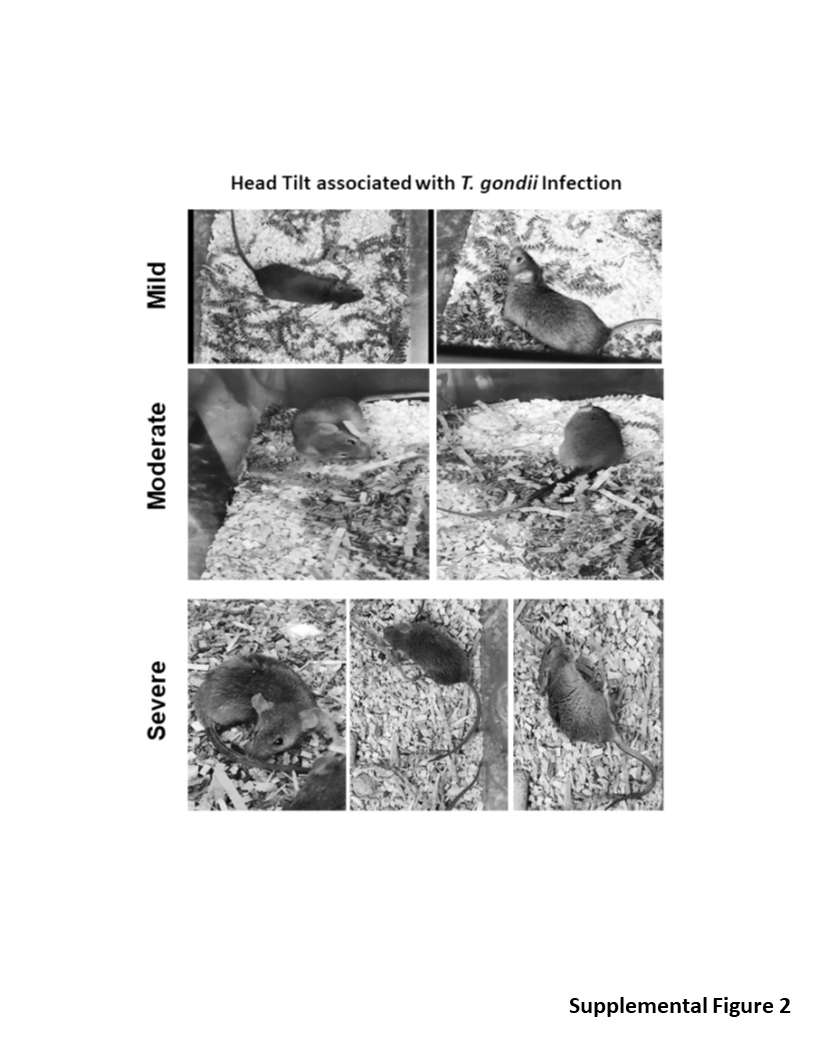

Supplement: Supplemental file 2 — Fig. S2. Download iai.00566-22-s0003.tif, TIF file, 0.4 MB [file iai.00566-22-s0003.tif]

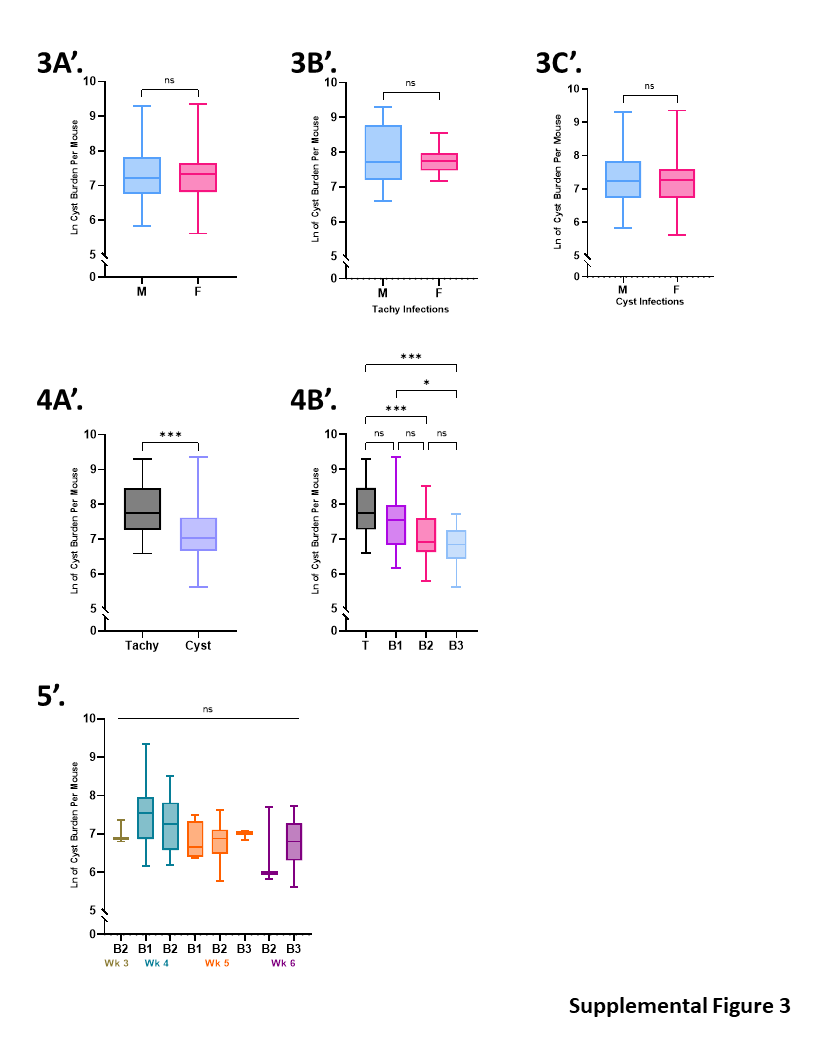

Supplement: Supplemental file 3 — Fig. S3. Download iai.00566-22-s0004.tif, TIF file, 0.09 MB [file iai.00566-22-s0004.tif]
