# Supplementary material for: Factors Influencing Tissue Cyst Yield in a Murine Model of Chronic Toxoplasmosis
Source: Infect Immun. 2023 Jun 26;91(7):e00566-22. doi: 10.1128/iai.00566-22 (PMC10353391; doi:10.1128/iai.00566-22)
Supplement: Supplemental file 5 — Legends of Fig. S1 to S3 and Movie S1. Download iai.00566-22-s0001.docx, DOCX file, 0.02 MB [file iai.00566-22-s0001.docx]

**Supplemental Data 1:**

**Supplemental File 1**

**Generation of the ME49ΔTgHXGPRT line**

Nucleotide scavenging pathway identifying the basis for the positive and negative selection associated with HXGPRT (HX) KO parasites. Parasites expressing HXGPRT are susceptible to 6-ThioXanthine (6-TX) but resistant to Mycophenolic Acid (MPA) + Xanthine. In contrast ΔHXGPRT parasites are resistant to 6-TX and sensitive to MPA-Xanthine. **B**. Schematic depicting the genomic locus encoding HXGPRT identifying the location of the PAM sites targeted with specific CRISPR-Cas9 constructs. **C**. Schematic of the genomic organization of the ΔHXGPRT locus with the sequence dendogram confirming the fusion of exons 2 and 5 due to the deletion of the intervening introns and exons resulting in the KO phenotype.

**D**. A plaque assay, reflecting the lytic cycle demonstrates that wild type ME49 parasites and the ME49ΔHXGPRT (HX) line exhibit so statistical differences for growth in cell culture. Plaques were randomly acquired and pooled from 3 independent experiments. A total of 51 WT ME49 and 51 ME49ΔHX plaques were measured. Analysis using unpaired 2-tailed t-test revealed no statistical differences.

**E**. Stage conversion monitored by intensity of fluorescent Dolichos lectin (DBA) staining in vitro, following exposure to alkaline pH8.2 media for 2, 4 and 6 days. reveals not statistical difference at each time point between the wild type ME49 and ME49ΔHX line.

A fixed number (15) of fields were captured at random and scored for each sample at each time point. A total of 40, 56, 35 ME49 (WT) vacuoles at days 2,4 and 6 and 60, 62 and 67 ME49ΔHXGPRT (HX) vacuoles at days 2,4 and 6 were analyzed. Pairwise analysis at each time point showed no difference in either the kinetics or extent of stage conversion.

**Supplemental Figure 2**

**Spectrum of head-tilt presentations in *Toxoplasma* infected mice**. A mild head tilt which either spontaneously resolves or progresses to moderate head tilt to a severe head tilt phenotype affecting motility and balance. Video recording of motility by head tilt presenting animals is provided in the Supplemental Movie SD4.

**Supplemental File 3**

Statistical analysis on Log(n) transformed data for data presented in Figure 3A,B,C, Figure 4A,B and Figure 5. While analysis of transformed data does not alter the significance for data in Figures 3 and 5, an increase in significance is observed for the effect of the inoculum (tachyzoite vs cyst) with the transformed data. L(n) transformation of the data in the parent Figure 4A exhibited higher significance in the cyst yields derived from tachyzoite and pooled bradyzoite infections (unpaired t-test, 2 tailed, P value = 0.0006, ***). In addition, related to parent Figure 4B increased significance was noted for T vs B2 (P value- 0.0010, ***) and T vs B3 (P value = 0.0004, ***). Additional significance was note for B1 vs B3 (P value= 0.0159, *).

**Supplemental File 4 (Video)**

**Recording of mild, moderate and severe head-tilt in *T. gondii* infected mice.**
